# Frail patients who fall and their risk on major bleeding and intracranial haemorrhage. Outcomes from the Fall and Syncope Registry

**DOI:** 10.1186/s12877-023-04120-9

**Published:** 2023-07-10

**Authors:** LAR Zwart, T Germans, R Vogels, S Simsek, MEW Hemels, RWMM Jansen

**Affiliations:** 1 Department of Geriatric Medicine, Northwest Clinics, Alkmaar, The Netherlands; 2 Aging and Public Health department, Amsterdam University Medical Centers, Amsterdam, The Netherlands; 3 Department of Geriatric Medicine, Dijklander Hospital, Hoorn, The Netherlands; 4 Department of Cardiology, Northwest Clinics, Alkmaar, The Netherlands; 5 Department of Neurology, Northwest Clinics, Alkmaar, The Netherlands; 6 Department of Internal Medicine, Northwest Clinics, Alkmaar, The Netherlands; 7 Department of Internal Medicine/Endocrinology, Amsterdam UMC, location VUmc, Amsterdam, The Netherlands; 8 Department of Cardiology, Rijnstate Hospital, Arnhem, The Netherlands; 9 Department of Cardiology, Radboud University Medical Centre, Nijmegen, The Netherlands; 10 Department of Cardiology, Amsterdam University Medical Centers, Amsterdam, The Netherlands

**Keywords:** Frailty, Cerebral small vessel disease, Major bleeding, Intra cranial haemorrhage, Oral anticoagulation

## Abstract

**Background:**

Major bleeding, and intracranial bleeding specifically, are severe complications related to the use of anticoagulation. To what extent the risk for major bleeding is elevated among frail older people is not well known because they are underrepresented in the randomized clinical trials (RCTs). This study investigates the risk for major bleeding (MB) and intra cranial haemorrhage (ICH) in frail older people who fall.

**Methods:**

All patients 65 years and older visiting the Fall and Syncope Clinic, between November 2011 and January 2020, and underwent a MRI of the brain were eligible. Frailty was assessed with a Frailty Index, based on the accumulation of deficits model. Cerebral small vessel disease was described and evaluated as proposed in the position paper of Wardlaw and colleagues in 2013.

**Results:**

479 patients were included in this analysis. Mean follow-up was 7 years per patient (ranging from 1 month to 8 years and 5 months). 368 patients (77%) were frail. A total of 81 patients used oral anticoagulation (OAC). 17 extracranial MB of which 3 were traumatic and 14 gastrointestinal, and 16 ICH occurred. There was a total of 603.4 treatment years with OAC, and 8 MBs occurred among patients on OAC (bleeding rate 1.32 per 100 treatment years), of which 2 ICHs (bleeding rate 0.33 per 100 treatment years). The risk for extracranial MB was increased by the use of antiplatelet agents (APA) (adjusted OR 6.9, CI 95% 1.2–38.3), and by the use of OAC (adjusted OR 9.8, CI 95% 1.7–56.1). The risk for ICH was only heightened by white matter hyperintensities (WMH) (adjusted OR 3.8, CI 95% 1.0-13.4). The use of APA (adjusted OR 0.9, CI 95% 0.3–3.3) or OAC (adjusted OR 0.6, CI 95% 0.1–3.3) did not elevate the risk for ICH.

**Conclusion:**

In contrast to common belief, frail patients on OAC with repeated falls show a comparable bleeding rate as in the large RCTs, and the use of OAC did not increase the risk for ICH. However, the number of MBs was low, and of ICHs very low, despite extensive follow-up in this registry.

**Supplementary Information:**

The online version contains supplementary material available at 10.1186/s12877-023-04120-9.

## Background

Geriatric patients are known to have multi morbidity, use multiple drugs, and to show cognitive disorders, mobility issues, and an increased risk of falling, which all lead to increasing frailty. This combination of physical and mental decline causes an older individual to become a geriatric patient, much more than old age alone [1–3]. Consequences of becoming frail are a heightened risk of suffering adverse events in general, such as hospitalisation, complications after medical procedures, delirium, and ultimately, death. The prevalence of atrial fibrillation (AF) and the concurrent use of anticoagulation is high among geriatric patients [4–6]. Furthermore, the prevalence of AF is expected rise in the coming decades [4]. And not only the risk of stroke, but also the risk of major bleeding (MB) increases with age [4]. At present, only a limited amount of data is available about the extent in which the risk for MB is heightened in frail patients, or by which factors specifically.

This study will investigate risk factors for MB, within a cohort of geriatric patients with repeated falls and or a syncope, as this normally presents as a fall. For many patients the evaluation of falls includes an MRI of the brain, as part of the assessment of a gait disorder, dementia diagnostics, suspicion of stroke, epilepsy, or other reasons. In this analysis, besides assessing the bleeding risk of known risk factors, geriatric features and cerebral vascular changes will be assessed as well.

## Methods

Inclusion and setting: The Fall and Syncope Registry periodically collects data on all patients who are assessed at the Fall and Syncope Clinic (FSC), starting in November 2011. The FSC is situated in a large teaching hospital facility, including neurosurgery and an intensive care unit. Details on the FSC have been published by Wold and colleagues [7]. To summarize, all patients undergo a Comprehensive Geriatric Assessment (CGA) that includes a medical, neurological, psychological, functional, and a cognitive evaluation. Only patients with multiple, unexplained falls are evaluated at the FSC, other patients who fell are assessed at the regular outpatient clinic and not included in the cohort or this analysis. Quality assurance of the data is periodically performed by RJ.

For this analysis, all consecutive patients aged 65 and older who visited the Fall and Syncope Clinic (FSC) from November 2011 until January 2020, and underwent an MRI scan of the brain as part of the FSC assessment were eligible. Data concerning bleeding outcomes, mortality and use of antithrombotic medication was retrospectively collected until January 2020. Since all patients repeatedly fall and are expected to be frail, the anticipated residual risk for an extracranial MB or intracranial haemorrhage (ICH) is anticipated to be high, for older people using oral anticoagulation (OAC), antiplatelet agents (APA) and also for those without antithrombotic medication. This gives the opportunity to calculate the additional risks for bleeding of various factors, in a cohort of frail patients that repeatedly fall. Data concerning major bleedings, mortality and the duration of antithrombotic treatment were retrieved from the electronic hospital files. The initial visit to the FSC was used for baseline data, and follow-up data was collected until January 2020, allowing for a variable follow-up period per patient. The duration of anticoagulation, with either Vitamin K antagonists (VKA) or non-vitamin K oral anticoagulation (NOAC), was retrieved from the electronic medical files. The duration was counted from initiation of OAC until a patient passed, until January 2020, or until a MB or ICH occurred. Years of use of OAC before visiting the FSC were included into the total amount of years on OAC, allowing for a duration of OAC use that is longer than the period that this study spans. Bleeding in a critical space (intracranial, intraspinal, intraocular, pericardial, intra-articular, retroperitoneal, or intramuscular with a compartment syndrome), bleeding leading to a decrease in the haemoglobin level of 20 g/L (1.24 mmol/L) or a fatal bleeding were considered as major bleeding episodes [8]. Haemorrhage in the brain tissue, subarachnoid haemorrhage, subdural and epidural bleedings were all considered ICH. Causes of death were retrieved from dismissal letters from the hospital, or checked at the general practitioners office if patients passed at home.

Patients were included in the analysis if an magnetic resonance image (MRI) of the brain had been performed as part of the FSC assessment, with suitable series to assess white matter hyperintensities (WMH), lacunes of presumed vascular origin (lacunes), cortical and hippocampal atrophy, and cerebral microbleeds (CMB). When describing these vascular changes, we applied the definitions and standards as proposed by Wardlaw and colleagues [9]. When assessing WMH, the Fazekas scale was applied [10]. Lacunes were counted based on their location: frontal, parietal or occipital, temporal, within the basal ganglia, or infratentorial. Cortical atrophy was graded using the Global Cortical Atrophy scale (GCA), and hippocampal atrophy using the meso temporal atrophy scale (MTA) [11]. The number of CMBs were counted, both in subcortical and in deep regions.

MRI details: All MR images were acquired with a 1.5 Tesla MRI machine. As proposed in the position paper by Wardlaw and colleagues in 2013, WMH, lacunes, and GCA were assessed on fluid-attenuated inversion recovery images (FLAIR), MTA was assessed on T1-weighted coronal images, and CMBs were assessed on susceptibility weighted images (SWI) [9].

Frailty details: Frailty was assessed by calculating a Frailty Index (FI) based on the accumulation of deficits model [2, 12]. The index comprised 34 items: 22 somatic items, 8 functional items, and 4 cognitive items. A list of items in the FI is presented in Supplementary Table 1. The FI ranges from 0 to 1, and patients were considered frail when being indexed 0.18 or higher, and severely frail with an index of 0.24 or higher [2, 6, 13].

Statistics: Analyses were performed using SPSS for Windows, version 20 (SPSS, Inc, Chicago, IL). Categorical variables were expressed as counts and percentages, continuous variables as mean values with standard deviation. Normality was assessed for continuous variables, e.g. age, FI, number of CMBs, and, if necessary, appropriate transformation was applied before further analysis. Odds Ratios (OR) with 95% confidence intervals (95% CI) were calculated, based on two-by-two tables. Regression analysis was performed to calculate the adjusted OR for non-ICH MB and ICH, using a multinomial logistic regression model. To construct the model for the type of MB as outcome, meaning non-ICH MB or ICH versus no MB, factors were used that had a statistically significant crude OR for bleeding, and a correction for age and frailty was applied.

## Results

**Fig. 1:**
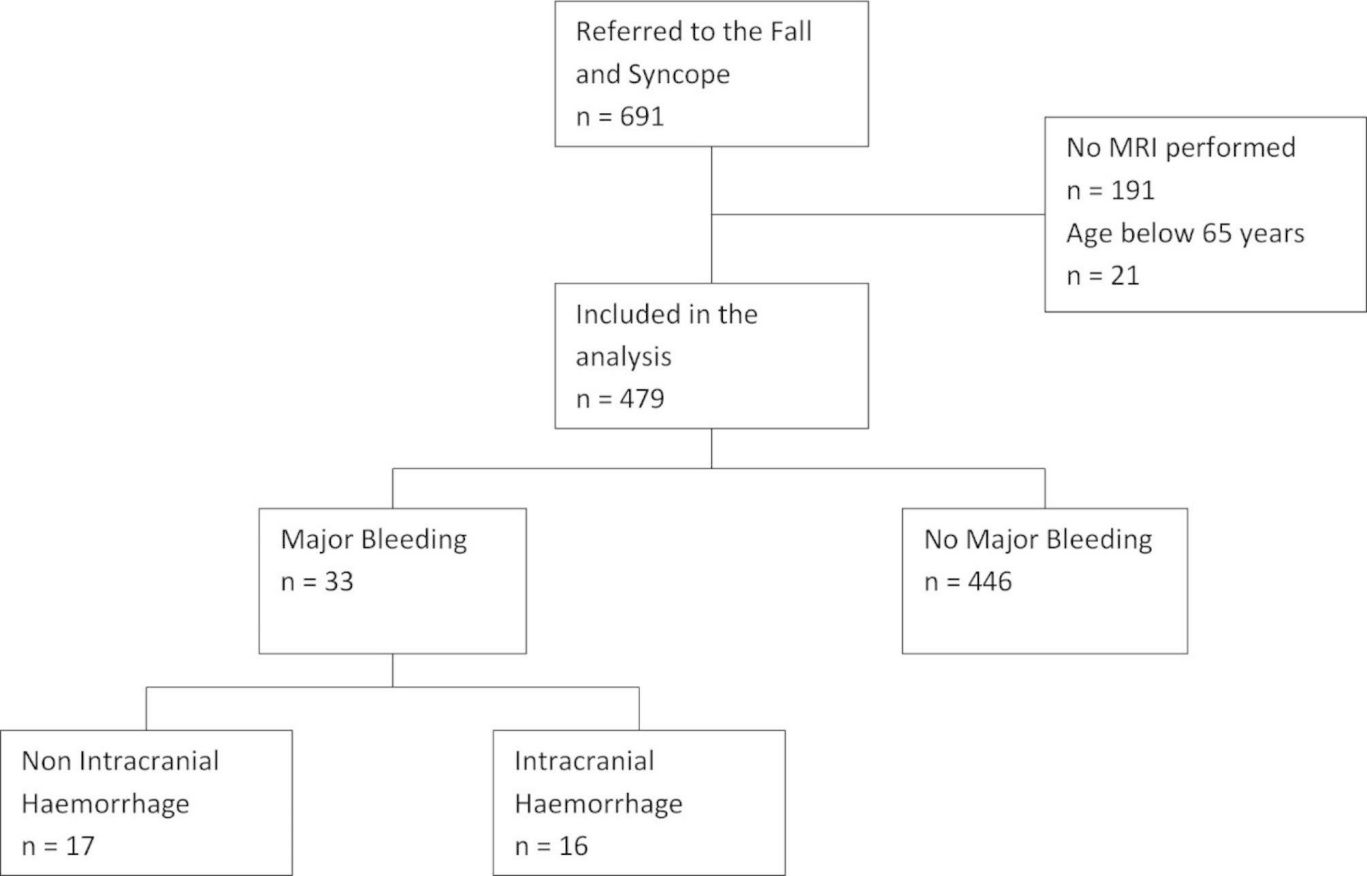
Study Flow Diagram

Between November 2011 and January 2020, a total of 691 patients visited the FSC, of whom 662 were aged 65 years and older. In 479 patients an MRI with all required series was performed. Figure 1 shows the inclusion process. Demographic data are presented in Table 1. The median age was 80 years (standard deviation (sd) 6.3 years), 326 patients were female (68%), the median number of drugs used was 7, polypharmacy was present in 337 patients (the use of 6 or more different drugs, 70%), and the median number of morbidities was 9. For patients with AF the median CHA_2_DS_s_Vasc score was 4 (sd 1.4) and median HASBLED score 3 (sd 1.1), corresponding an expected rate of bleeding in between 1.88 and 3.74% per year. Between November 2011 and January 2020, 138 patients died (28.8%). The five most prevalent morbidities were hypertension (65%), gait disturbances (52%), post prandial and orthostatic hypotension (29% and 33%), diabetes mellitus (22%), and AF (21%). A total of 248 patients used antithrombotic medication, 166 used APA (36%), 81 OAC (17%) and 1 patient used both. In total, there were 603.4 patient years of treatment with OAC, counted from the moment patients started using OAC, until the first of January 2020 or until they stopped using OAC. The mean duration of OAC treatment was 7.0 years, with a range of 7 months to 21 years. The mean frailty index was 0.22 (sd 0.09), 128 patients were frail (26.7%) and 240 patients were severely frail (50.1%). Patients without an MRI had a median age of 80 years and used a median of 7 prescription drugs. They had a slightly higher average CHA_2_DS_2_Vasc score of 4.0 (students T test, *p* 0.05) and a higher average Frailty Index of 0.25 (students T test, *p* < 0.01). A higher proportion was known with AF (31.7%, *p* < 0.01) and were iADL dependent (48.1%, *p* 0.01). A detailed description of the patients characteristics is presented in Supplementary Table 2. In summary, patients who did not undergo a MRI were frailer, had more morbidities, used more medication and were more dependent on other for daily activities.

**Table 1:** Baseline characteristics

General characteristics	Total, n = 479
Female, n (%)	326 (68.1)
Age in years, median (sd)	80 (6.3)
Major bleeding	
Extracranial major bleeding, n (%)	17 (3.5)
Intracranial haemorrhage (ICH), n (%)	16 (3.3)
Number of drugs, median (sd)	6 (3.7)
Number of morbidities, median (sd)	9 (5.0)
Multiple falls per year, n (%)	430 (91.1)
Atrial fibrillation, n (%)	100 (20.9)
Hypertension, n (%)	312 (65.1)
Diabetes mellitus, n (%)	107 (22.3)
Chronic Kidney Disease*, n (%)	67 (14)
Heart failure, n (%)	40 (8.4)
Stroke in medical history, n (%)	97 (20.3)
CHA2DS2VASC, median (sd) for patients with atrial fibrillation (n = 100)	4 (1.4)
HASBLED, mean (sd) for patients with atrial fibrillation (n = 100)	3 (1.1)
Use of OAC, n (%)	81 (16.9)
Use of APA, n (%)	166 (34.7)
Geriatric features	
Frailty Index, mean (sd)	0.22 (0.09)
Moderate Frailty, n (%)	128 (26.7)
Severe Frailty, n (%)	240 (50.0)
Polypharmacy**, n (%)	337 (70.4)
Orthostatic hypotension, n (%)	140 (29.2)
Post prandial hypotension, n (%)	154 (32.9)
Parkinsonism, n (%)	44 (9.2)
Gait disturbance, n (%)	250 (52.2)
ADL dependence, n (%)	104 (21.7)
iADL dependence, n (%)	174 (36.3)
Cognitive impairment	
MMSE < 26 points, n (%)	120 (25.1)
MoCA < 26 points, n (%)	281 (58.7)
Dementia in medical history, n (%)	24 (5.0)
MRI findings	
Number of cerebral microbleeds, mean, median (sd)	1.8, 0 (6.9)
Presence of cerebral microbleeds, n (%)	169 (35.8)
Presence of lacunes, n (%)	115 (24.1)
Fazekas score, mean (sd)	1.8 (0.9)
Fazekas score ≥ 2, n (%)	253 (52.9)
Macro infarction, n (%)	42 (8.8)
MTA score, mean (sd)	2.0 (1.5)
Relevant MTA, n (%)	281 (58.8)
Global atrophy, mean (sd)	1.4 (0.8)

Abbreviations: OAC oral anticoagulation, APA antiplatelet agents, ADL activities of daily living, iADL instrumental activities of daily living, MMSE Mini Mental State Examination, MoCA Montreal Cognitive Assessment, MTA mesotemporal atrophy.

The differences between non-frail, moderately frail and severely frail patients are described in Supplementary Table 3. In summary, with increasing frailty the number of prescription drugs increased as well from 3 to 8, the proportion of all relevant morbidities and polypharmacy increased, a higher score on the CHA_2_DS_2_Vasc and HASBLED was present. Furthermore, the proportion of dependency in activities of daily living (ADL) or instrumental activities of daily living (iADL) was higher, and cognitive impairment more prevalent. Patients that were either frail or severely frail did not have a higher occurrence of major and intra cranial bleeding than non-frail patients. There was no difference in the number of CMBs, lacunes, and Fazekas score, between frail and non-frail patients. No patients were lost to follow up.

**Fig. 2:**
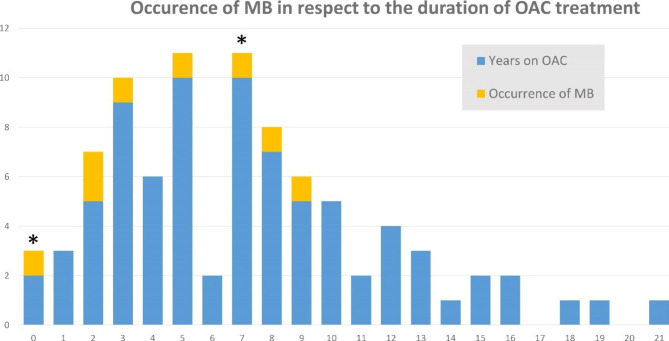
Occurrence of Major Bleeding or Intracranial Haemorrhage in relation to OAC duration

Between November 2011 and January 2020, a total of 33 MBs occurred: namely 16 ICH, 3 traumatic and 14 gastrointestinal. The three traumatic bleedings consisted of 2 subcutaneous bleedings in the lower extremity, and one retroperitoneal bleeding because of a pelvic fracture. All three patients used Vitamin K antagonists. None of the ICH were the consequence of a fall. Of all major bleedings, 16 (48%) occurred in patients using APA, and 8 occurred in patients on OAC (24%), 2 ICH occurred in patients on OAC (12.5% of all ICH). There were 1.32 MBs and 0.33 ICHs per 100 treatment years of OAC. Figure 2 show the distribution of the duration of OAC use, and the occurrence of MB and ICH, the asterix indicates the occurence of an ICH.

Table 2 presents the risks for extracranial MB and ICH. For patients with an extracranial MB, the HAS-BLED score was 3.0, and without 2.8, *p* 0.31. For patients with ICH, the HAS-BLED score was 2.7, and without ICH 2.8, *p* 0.71. Frailty was not associated with a risk for either extracranial MB or ICH. A higher risk for extracranial MB was found in the crude analysis for AF, ischemic stroke in the medical history, and use of APA. The use of OAC has an OR of 2.8, but this was not a statistically significant effect (95% CI 0.99–7.77). For ICH, only an association with WMH (defined as a Fazekas score of 2 points or higher) was found. A small number of patients had already been diagnosed with dementia before visiting the FSC (24 patients, 5.0%), and in these patients the risk for both MB and ICH were not significantly elevated. Cognitive screening, however, indicated that many more patients had a cognitive impairment (abnormal Mini Mental State Examination (MMSE) in 120 patients, 25%, and abnormal Montreal Cognitive Assessment (MoCA) in 281 patients, 59%). Among the patients with an abnormal MMSE, there was a non-statistically significant elevation of the risk for MB, and for ICH (OR 2.2, CI 95% 0.8-6.0, and OR 2.5, CI 95% 0.9–6.8, respectively).

The outcomes of the multinomial logistic regression are shown in Table 3. Because of collinearity between atrial fibrillation and the use of OAC, atrial fibrillation was not entered into the model. For extracranial MB after adjustment, the use of either APA or OAC were clearly associated with an elevated risk for bleeding, with very wide confidence intervals (APA: OR 6.9, CI 95% 1.24–38.32, OAC OR 9.8 CI 95% 1.70–56.10), and a history of ischemic stroke was no longer associated (OR 2.7, CI 95% 0.96–7.82). For ICH after adjustment, WMH remained a risk for intracranial bleeding with a comparable magnitude and confidence interval as in the crude analysis (OR 3.75, CI 95% 1.03–13.67).

Between November 2011 and January 2020, a total of 138 patients (28.8%) died. The causes of death were infection (23 patients, 4.8%), malignancy (14 patients, 2.9%), major cardiovascular events (heart failure 10 patients 2.1%, dissection 1 patient 0.2%, ruptured aneurysm 1 patient 0.2%), major bleeding (8 patients 1.7%), traumatic injuries (5 patients, 1.0%), stroke (5 patients, 1.0%), end stage chronic obstructive pulmonary disease (2 patients, 0.4%), and a majority of patients passed at home and no reliable assessment of the cause of death was available (69 patients, 12.7%). The patients that died during follow up had a higher age at baseline (82 versus 79, p < 0.001), a higher FI (0.26 versus 0.21, p < 0.001), were more often known with heart failure (52.5% vs. 26.7%, p 0.001). Mortality was strongly associated with frailty (OR 2.7, CI 95% 1.4 to 5.4), severe frailty (OR 4.0, CI 95% 2.2 to 7.4), and heart failure (OR 3.0, CI 95% 1.6 to 5.9).

**Table 2:** Crude risks for non-ICH MB and ICH

	Non intracranial haemorrhage			Intra cranial haemorrhage		
Factor	non-ICH MB, n = 17	Crude OR non-ICH MB	95% CI	ICH, n = 16	Crude OR ICH	95% CI
Gender (female), n (%)	12 (70.6)	1.13	0.39–3.28	11 (68.8)	1.04	0.35–3.04
Atrial fibrillation, n (%)	7 (41.2)	2.73	1.01–7.37	2 (12.5)	0.56	0.12–2.50
Hypertension, n (%)	13 (76.5)	1.8	0.58–5.62	11 (68.8)	1.22	0.42–3.57
Chronic Kidney Disease*, n (%)	1 (5.9)	0.37	0.05–2.81	1 (6.3)	0.39	0.05–3.01
Diabetes mellitus, n (%)	4 (23.5)	1.09	0.35–3.43	4 (25.0)	1.18	0.37–3.75
Ischemic stroke in medical history, n (%)	9 (52.9)	4.85	1.82–12.94	4 (25.0)	1.44	0.45–4.57
Heart failure, n (%)	3 (17.6)	2.44	0.67–8.89	1 (6.3)	0.76	0.10–5.91
Use of OAC, n (%)	6 (35.3)	2.79	0.99–7.77	2 (12.5)	0.73	0.16–3.28
Use of APA, n (%)	10 (58.8)	2.88	1.07–7.71	6 (37.5)	1.21	0.43–3.39
Moderate Frailty, n (%)	5 (29.4)	1.52	0.36–6.52	7 (43.8)	1.4	0.37–5.28
Severe Frailty, n (%)	9 (52.9)	2.13	0.54–8.45	6 (37.5)	0.93	0.23–3.80
Polypharmacy**,. n (%)	15 (88.2)	3.19	0.72–14.1	8 (50.0)	0.43	0.16–1.16
Orthostatic hypotension, n (%)	4 (23.5)	1.08	0.26–4.39	6 (37.5)	1.29	0.39–4.33
Post prandial hypotension, n (%)	6 (35.3)	5.2	0.62–43.77	7 (43.8)	2.02	0.51–7.99
Parkinsonism, n (%)	1 (5.9)	0.6	0.08–4.65	2 (12.5)	1.37	0.30–6.25
Gait disturbance, n (%)	7 (41.2)	0.62	0.23–1.67	8 (50.0)	0.89	0.33–2.41
ADL dependence, n (%)	3 (17.6)	0.75	0.21–2.67	3 (18.8)	0.81	0.23–2.90
iADL dependence, n (%)	6 (35.3)	0.96	0.35–2.63	6 (37.5)	1.05	0.38–2.95
Cognitive impairment, n (%)						
–MMSE < 26 points	7 (41.2)	2.24	0.83–6.03	7 (43.8)	2.49	0.91–6.84
–MoCA < 26 points	9 (52.9)	0.78	0.29–2.05	10 (62.5)	1.15	0.41–3.22
Dementia in medical history, n (%)	1 (5.9)	4.5	0.51–39.6	1 (6.3)	5.1	0.58–45.6
Presence of cerebral microbleeds, n (%)	5 (29.4)	0.82	0.28–2.41	6 (37.5)	1.21	0.42–3.45
Presence of lacunes, n (%)	3 (17.6)	0.69	0.19–2.44	7 (43.8)	2.5	0.91–6.86
Fazekas score ≥ 2, n (%)	11 (64.7)	1.72	0.63–4.74	13 (81.3)	4.07	1.14–14.48
Relevant MTA, n (%)	10 (58.8)	1.03	0.38–2.75	12 (75.0)	2.15	0.68–6.79

## Discussion

Physicians are concerned about the risk of ICH because of anticoagulation, especially among those elderly patients who frequently fall [14, 15]. Dutch guidelines warrant caution when prescribing NOAC [16]. However, data on bleeding risks associated with falling are scarce. Well-known risk factors for ICH are age, previous strokes and bleeding, hypertension, renal impairment, diabetes mellitus and dementia [4, 17]. Cerebral microbleeds (CMB) might also increase the risk of ICH. The CROMIS 2 study showed an increased risk for ICH in patients with AF, recent strokes, or transient ischemic attacks (TIA), and at least 1 CMB at the time of inclusion [18]. However, another cohort of patients with AF and at least 1 CMB, with or without a previous stroke or TIA at baseline, did not show a higher incidence of MB among patients using warfarin or a NOAC, compared with patients without anticoagulation, despite high CHA_2_DS_2_Vasc scores and HAS-BLED score in all study arms (6 and 5 points respectively) [19].

This study investigated factors associated with major bleeding, and intra cranial haemorrhage, among a cohort of geriatric patients with repeated falls. On average, this cohort was severely frail, and 90% frequently fell. Patients had a median age of 80 years, suffered from multi morbidity and polypharmacy. Consequently, this is a cohort with a high anticipated bleeding risk, reflected in the HAS-BLED score of 2.8. Our study shows two important findings.

Firstly, the number of MBs and ICHs in patients on OAC is low, with 1.32 MB and 0.33 ICH per 100 patient treatment years, a bleeding rate comparable to, or lower than, the rates found in the large RCTs [20–24]. In this cohort most patients used VKA (1 patient was on a NOAC and did not experience a bleeding event). In the AMADEUS trial the rate of MB in the warfarin arm was 2.6 [20], the RE-LY study 3.4 [21], the ARISTOTLE trial 3.1 [22], the ENGAGE-AF-TIMI 3.4 [23], and in the ROCKET-AF 3.4 MB per 100 treatment years [24]. The average HASBLED score of our cohort was 2.8, which is comparable to the ROCKET-AF trial, but the bleeding event rate was much lower. A possible explanation for this difference lies in our extensive follow-up. The trials vary between 1 [20] and 2.8 [21] years of follow-up per patient, whereas in our study twenty-two out of 81 (27%) patients used OAC for more than 10 years. The bleedings in our cohort occurred during the range of 7 months to 10 years of OAC use, well beyond the duration of follow-up of the RCTs. This results in a much higher total number of treatment years and therefore a lower ratio of bleeding per 100 years. Simultaneously, among patients who had used OAC for over 10 years, no bleedings occurred. Our data could suggest that if a patient is prone to bleeding, bleeding will occur relatively early. Another possible explanation could lie within competing factors. A large proportion of the cohort died during the follow up, 29%, as would be expected for this age group. Since the majority of patients were frail, in general a higher rate of mortality and complications is to be expected [1–3]. This could have led to patients dying of other causes, instead of suffering a MB, and a lower rate of bleeding per 100 treatment years.

The second important finding is that frailty does not seem to raise the risk for extracranial MB or ICH. Most patients in this cohort were frail or severely frail at baseline. As to be expected, frail patients had a high mortality rate (non-frail 12.6%, frail patients 28.1%, severely frail 36.7%). Frailty was however not associated with either extracranial MB or ICH. Of cerebral small vessel disease, WMH are associated with ICH (adjusted OR 3.75, CI 95% 1.03–13.67), but CMB, and lacunes, were not. Also, global cortical atrophy or mesotemporal atrophy were not associated with an increased risk for ICH. In this cohort, the well-known risk factors for ICH were not associated with an increased risk for ICH. An explanation could be that the patients within this cohort have such a high prevalence of those classic risk factors, that they thereby might have lost their discriminating properties.

The MoCA was designed to detect mild cognitive impairment (MCI) [25] and is also sensitive to vascular cognitive impairment [26], while the MMSE is generally used as a screening tool for Alzheimer’s dementia [27]. The MMSE contains fewer complex items, and, therefore, if patients should score abnormally, this could reflect a more severe cognitive decline when compared to the MoCA. The difference in being at risk for major bleeding between patients with a low score on the MMSE or MoCA, which was observed within this cohort, is therefore relevant in two ways. Firstly, it suggests that as the cognitive decline becomes more severe, the risk of ICH seems to increase, although our data are not suitable to ascertain how this risk develops, and more power is needed for a better estimation of the magnitude of that risk. Secondly, it could be relevant to incorporate cognitive screening into the general assessment of patients if OAC is initiated. Regarding patients with an abnormal score on the MMSE, it could be considered to consult a geriatrician and perform a CGA, including an assessment of polypharmacy, thus enabling a multidisciplinary approach to the continuation, initiation, or discontinuation of anticoagulation.

When taking the severity of frailty within this cohort into consideration, the results described above do not support the assumption that frail older people run an excessive risk of suffering anticoagulation related bleedings as compared to the fitter older people who were studied in the large RCTs. On the contrary, our findings support the latest ESC guideline on AF that emphasizes a low absolute risk for MB including ICH, and that the benefits of OAC outweigh the risk of bleeding [4]. Since the anticoagulation used in this cohort mostly consists of VKA (80 out of 81, 98.8%), the safety profile could even be better for patients on NOACs [4]. The results of this study, however, show that caution to prescribe OAC to frail older people might be unnecessary and could lead to withholding treatment for those patients at the highest risk of suffering a stroke.

Our study has limitations. The observational design of the study only allows a descriptive analysis and can give an indication of the risks for MB, but cannot provide as robust evidence as a randomized clinical trial can. Furthermore, due to the low number of bleeding evets, the extent to which adjusted Odds Ratios could be calculated is limited. However, the adjusted OR’s are comparable to the crude OR’s, with a substantial increase in the size of the 95% confidence intervals for the risk on extracranial MB of using either APA or OAC, and a slight narrowing of the confidence interval for the risk for ICH associated with WMH. The OR’s were adjusted for the factors with a statistically significant crude OR, except for AF because collinearity with OAC, and age and frailty were added to the model. The adjusted OR’s indicate that, naturally, the use of either APA or OAC increases the risk for major bleeding, but in absolute numbers this results in a bleeding rate comparable to the bleeding rate within the large RCTs [20–24]. Our findings do not support the assumption that frail older people who fall are at a higher risk for major bleeding.

Another limitation is that there was no reassessment of frailty and other medical conditions during follow up. Frailty is a dynamic concept and gain of functionality is possible. Frailty increases over time, it possible that patients who were not frail at baseline became frail during follow up [2]. Within this analysis it was not possible to take an increase in frailty over time into account. Also, detailed information concerning medication adherence, blood pressure, cholesterol, renal function, and diabetes mellitus regulation during the follow-up was not available.

**Table 3:** Adjusted risks for non-ICH MB and ICH

Multinomial logistic regression model		
Non-ICH MB	Adjusted OR	95% CI
Age	1.03	0.94–1.13
Ischemic stroke in medical history	2.74	0.96–7.82
Use of OAC	9.78	1.70–56.10
Use of APA	6.9	1.24–38.32
Moderate Frailty	0.64	0.16–2.60
Severe Frailty	0.94	0.20–4.35
Fazekas score 2 or higher	1.18	0.41–3.40
ICH	Adjusted OR	95% CI
Age	1.06	0.97–1.15
Ischemic stroke in medical history	1.35	0.35–5.16
Use of OAC	0.63	0.12–3.26
Use of APA	0.93	0.26–3.34
Moderate Frailty	0.76	0.17–3.32
Severe Frailty	1.8	0.44–7.51
Fazekas score 2 or higher	3.75	1.03–13.67

Abbreviations: MB Major Bleeding, OAC oral anticoagulation. Footnote: * indicates that the Major Bleeding is an intracranial haemorrhage

## Conclusion

Within this cohort of frail patients assessed for repeated falls, a bleeding rate comparable to the large RCTs was observed, and the use of antithrombotic medication did not heighten the risk for ICH. The number of MBs was low, and of ICHs very low, despite extensive follow-up. In this study, cerebral microbleeds were not seen to elevate the risk of ICH, but WMH were associated with ICH. Interestingly, a low score on the MMSE could be associated with ICH. We therefore encourage physicians to perform cognitive screening of all patients with a substantial risk for suffering from cardiovascular events. Based on the findings of this study, we strongly encourage further research into OAC related ICH among high-risk patients to focus on cerebral small vessel disease such as MWH, and to focus on cognitive decline.

## Electronic supplementary material

Below is the link to the electronic supplementary material.

Supplementary Material 1

Supplementary Material 2

Supplementary Material 3

## Data Availability

The datasets generated and/or analysed during the current study are not publicly available because data sharing was not within the patients informed consent procedure, but selections of data are available from the corresponding author upon reasonable request.

## List of Abbreviations

AF: Atrial fibrillation
Extracranial MB: Extracranial Major bleeding
ICH: Intracranial haemorrhage
NOAC: Non-vitamin K oral anticoagulation
CMB: Cerebral microbleeds
CHA_2_DS_2_Vasc: Congestive Heart Failure, Hypertension, Age ≥ 75, Diabetes, Stroke/ TIA/ Thromboembolism, Vascular disease, Age 65–74, Female
HAS-BLED: Hypertension, Abnormal renal or liver function, Stroke, Bleeding, Labile INR, Elderly (≥ 65), Drugs or alcohol
TIA: Transient ischemic attack
FSC: Fall and Syncope Clinic
CGA: Comprehensive Geriatric Assessment
VKA: Vitamin K antagonist
MRI: Magnetic resonance imaging
WMH: White matter hyperintensities
GCA: Global cortical atrophy
MTA: Mesotemporal atrophy
FLAIR: Fluid-attenuated inversion recovery
SWI: Susceptibility weighted images
FI: Frailty Index
OR: Odds Ratio
95% CI: 95% confidence interval
APA: Anti platelet agents
OAC: Oral anticoagulation
ADL: Activities of daily living
iADL: Instrumental activities of daily living
MMSE: Mini Mental State Examination
MoCA: Montreal Cognitive Assessment
