# Supplementary material for: Frail patients who fall and their risk on major bleeding and intracranial haemorrhage. Outcomes from the Fall and Syncope Registry
Source: BMC Geriatr. 2023 Jul 10;23:422. doi: 10.1186/s12877-023-04120-9 (PMC10334580; doi:10.1186/s12877-023-04120-9)
Supplement: Supplementary file 1 — Supplementary Material 1 [file 12877_2023_4120_MOESM1_ESM.docx]

**Supplementary Table 1. Items in the Frailty Index**

Atrial Fibrillation

Mild Cognitive Impairment

Dementia

Syncope

Heart Failure

Ischemic Heart Disease

Peripheral arterial disease

Stroke

Multiple Stroke

Diabetes Mellitus

Liver cirrhosis

Hypertension

Anaemia

Renal Impairment, class 2 or up

Electrolyte abnormality

Hypothyroidism

Hyperthyroidism

Subclinical Hypothyroidism

Subclinical Hyperthyroidism

Malignancy

Epilepsy

Schizophrenia

Morbus Parkinson or parkinsonism of other causes

Gait disorder

Visual Impairment

Auditory Impairment

Osteoporosis

Arthrosis

Chronic Obstructive Pulmonary Disease

Asthma

Obstructive Sleep Apnoea Syndrome

Mood disorder

Anxiety disorder

Falls

Obesitas

Underweight

Gout

Polyneuropathy

Polypharmacy

MMSE below 26 points

MoCA below 26 points

MNA lower than 12 points

ADL dependent

iADL dependent

Social status (living alone)

Explanatory note: All factors are assigned an equal weight if present in the patient. The index is calculated as the sum of factors, divided by 44. Polypharmacy is defined as using 6 or more different drugs.

Abbreviations. MMSE: Mini Mental State Examination, MoCA: Montreal Cognitive Assessment, MNA: Mini Nutritional Assessment, ADL: Activities of Daily Living, iADL: instrumental Activities of Daily Living.
