# Supplementary material for: Frail patients who fall and their risk on major bleeding and intracranial haemorrhage. Outcomes from the Fall and Syncope Registry
Source: BMC Geriatr. 2023 Jul 10;23:422. doi: 10.1186/s12877-023-04120-9 (PMC10334580; doi:10.1186/s12877-023-04120-9)
Supplement: Supplementary file 2 — Supplementary Material 2 [file 12877_2023_4120_MOESM2_ESM.docx]

| **Supplementary Table 2.** |  |  |  |
| --- | --- | --- | --- |
|  |  |  |  |
|  | **With MRI, (n=479)** | **Without MRI, (n=183)** | ***p*** |
| Age in years, median (sd) | 80 (6,3) | 80 (6,9) | 0,38 |
| Number of drugs, median (sd) | 6 (3,7) | 7 (3,8) | <0,01 |
| Number of morbidities, median (sd) | 9 (5,0) | 10 (5,5) | <0,01 |
| BMI, mean (sd) | 26,4 (4,3) | 26,6 (4,6) | 0,4 |
| Kidney function, eGFR mL/min, mean (sd) | 55,5 (8,5) | 56,0 (8,2) | 0,73 |
| CHA2DS2VASC, mean (sd) | 3.8 (1.4) | 4,0 (1,4) | 0,05 |
| HASBLED, mean (sd) | 2.8 (1.1) | 2,8 (1,2) | 0,69 |
| Frailty Index, mean (sd) | 0,22 (0,09) | 0,25 (0,10) | <0,01 |
| MNA score, mean (sd) | 12,0 (2,2) | 12,1 (2,1) | 0,2 |
| MMSE score, mean (sd) | 26,0 (3,8) | 26,7 (3,2) | 0,04 |
| MoCA score, mean (sd) | 23,4 (4,1) | 23,3 (4,0) | 0,38 |
| Female, n (%) | 326 (68,1) | 110 (60,1) | 0,06 |
| Died | 128 (26,7) | 57 (31,1) | 0,29 |
| Multiple falls per year, n (%) | 430 (91,1) | 153 (83,6) | 0,03 |
| Major Bleeding after Fall Syncope clinic |  |  |  |
| Non ICH, n (%) | 17 (3,5) | 3 (1,6) | 0,18 |
| Intracranial haemorrhage, n (%) | 16 (3,3) | 10 (5,5) | 0,23 |
| Atrial fibrillation, n (%) | 100 (20,9) | 58 (31,7) | <0,01 |
| Hypertension, n (%) | 312 (65,1) | 120 (65,6) | 0,88 |
| Diabetes mellitus, n (%) | 107 (22.3) | 42 (23,0) | 0,82 |
| Chronic Kidney Disease*, n (%) | 67 (14,0) | 22 (12,0) | 0,51 |
| Heart failure, n (%) | 40 (8,4) | 25 (13,7) | 0,06 |
| Stroke in medical history, n (%) | 97 (20,3) | 52 (28,4) | 0,02 |
| Prior myocardiac infarct, n (%) | 52 (10,4) | 26 (14,2) | 0,23 |
| Use of OAC, n (%) | 81 (16,9) | 59 (32,2) | <0,01 |
| Use of APA, n (%) | 166 (34.7) | 59 (32,2) | 0,65 |
| Chronic Obstructive Pulmonary Disease, n (%) | 90 (18,8) | 40 (21,9) | 0,38 |
| History of malignancy, n (%) | 113 (23,6) | 51 (27,9) | 0,27 |
| Osteoporosis, n (%) | 71 (14,8) | 30 (16,4) | 0,63 |
| Hearing impairment, n (%) | 106 (22,2) | 51 (28,2) | 0,12 |
| Visual impairment, n (%) | 204 (42,8) | 82 (44,8) | 0,66 |
| Polypharmacy**, n (%) | 337 (70.4) | 147 (80,3) | 0,01 |
| Orthostatic hypotension, n (%) | 140 (29,2) | 54 (29,5) | 1 |
| Post prandial hypotension, n (%) | 154 (32,9) | 53 (29,0) | 0,45 |
| Parkinsonism, n (%) | 44 (9.2) | 17 (9,3) | 1 |
| Gait disturbance, n (%) | 250 (52.2) | 94 (51,4) | 0,86 |
| Walking aid, n (%) | 265 (55,3) | 100 (54,9) | 0,82 |
| ADL dependence, n (%) | 104 (21.7) | 52 (28,4) | 0,08 |
| iADL dependence, n (%) | 174 (36.3) | 88 (48,1) | 0,01 |
| Dementia in medical history, n (%) | 24 (5,0) | 14 (7,7) | 0,2 |
|  |  |  |  |
| *Binary logistic regression: as compared to no Major Bleeding | |  |  |
| **6 prescription drugs or more |  |  |  |
