# Supplementary material for: Frail patients who fall and their risk on major bleeding and intracranial haemorrhage. Outcomes from the Fall and Syncope Registry
Source: BMC Geriatr. 2023 Jul 10;23:422. doi: 10.1186/s12877-023-04120-9 (PMC10334580; doi:10.1186/s12877-023-04120-9)
Supplement: Supplementary file 3 — Supplementary Material 3 [file 12877_2023_4120_MOESM3_ESM.docx]

| **Supplementary Table 3. Baseline characteristics per frailty category** | | |  |
| --- | --- | --- | --- |
|  |  |  |  |
| General characteristics | Not frail, n=111 | Moderate Frailty, n=128 | Severe Frailty, n=240 |
| Female, n (%) | 75 (67,6) | 84 (65,6) | 167 (69,6) |
| Age in years, median (sd) | 78 (6,1) | 81 (6,3) | 81 (6,2) |
| Major bleeding |  |  |  |
| Non-ICH major bleeding, n (%) | 3 (2,7) | 5 (3,9) | 9 (3,8) |
| Intracranial haemorrhage (ICH), n (%) | 3 (2,7) | 7 (5,5) | 6 (2,5) |
| Number of drugs, median (sd) | 3 (2,9) | 6 (2,9) | 8 (3,6) |
| Number of morbidities, median (sd) | 7 (3,6) | 10 (4,5) | 12 (5,5) |
| Multiple falls per year, n (%) | 91 (82,0) | 114 (89,1) | 225 (93,0) |
| Atrial fibrillation, n (%) | 13 (11,7) | 20 (15,6) | 67 (27,9) |
| Hypertension, n (%) | 49 (44,1) | 79 (61,7) | 183 (76,2) |
| Diabetes mellitus, n (%) | 11 (9,9) | 25 (19,5) | 70 (29,2) |
| Chronic Kidney Disease*, n (%) | 4 (3,6) | 13 (10,2) | 50 (20,8) |
| Heart failure, n (%) | 0 (0,0) | 10 (7,8) | 30 (12,5) |
| Stroke in medical history, n (%) | 10 (9,0) | 27 (21,1) | 60 (25,0) |
| CHA2DS2VASC, mean (sd) | 3,2 (1,2) | 3,9 (1,2) | 4,6 (1,4) |
| HASBLED, mean (sd) | 2,4 (1,1) | 2,7 (1,1) | 3,1 (1,2) |
| Use of OAC, n (%) | 11 (9,9) | 18 (14,1) | 52 (21,7) |
| Use of APA, n (%) | 16 (14,4) | 44 (34,4) | 104 (43,3) |
| Geriatric features |  |  |  |
| Frailty Index, mean (sd) | 0,13 (0,04) | 0,22 (0,01) | 0,31 (0,05) |
| Polypharmacy**, n (%) | 27 (24,3) | 92 (71,9) | 217 (90,4) |
| Orthostatic hypotension, n (%) | 18 (31,6) | 24 (35,6) | 99 (59,3) |
| Post prandial hypotension, n (%) | 24 (45,3) | 35 (53,8) | 96 (58,5) |
| Parkinsonism, n (%) | 5 (4,5) | 7 (5,5) | 33 (13,8) |
| Gait disturbance, n (%) | 36 (32,4) | 61 (47,7) | 154 (64,2) |
| ADL dependence, n (%) | 6 (5,4) | 20 (15,6) | 79 (32,9) |
| iADL dependence, n (%) | 15 (13,5) | 37 (28,9) | 122 (50,8) |
| Cognitive impairment |  |  |  |
| MMSE < 26 points, n (%) | 18 (16,2) | 32 (25,0) | 70 (29,3) |
| MoCA < 26 points, n (%) | 51 (46,4) | 76 (59,4) | 154 (64,7) |
| Dementia in medical history, n (%) | 2 (1,8) | 4 (3,1) | 11 (4,6) |
| MRI findings |  |  |  |
| Number of cerebral microbleeds, mean, median (sd) | 0 (9,3) | 0 (2,6) | 0 (6,3) |
| Presence of cerebral microbleeds, n (%) | 35 (31,5) | 47 (36,7) | 86 (35,8) |
| Presence of lacunes, n (%) | 24 (21,6) | 35 (27,3) | 57 (23,8) |
| Fazekas score, mean (sd) | 1,7 (0,9) | 1,7 (0,9) | 1,9 (0,9) |
| Fazekas score ≥ 2, n (%) | 51 (45,9) | 67 (52,3) | 136 (56,7) |
| Macro infarction, n (%) | 0 (0,0) | 14 (10,9) | 28 (11,7) |
| MTA score, mean (sd) | 1,8 (1,5) | 2,0 (1,6) | 2,1 (1,6) |
| Relevant MTA, n (%) | 59 (53,2) | 66 (52,0) | 156 (65,0) |
| Global atrophy, mean (sd) | 1,1 (0,7) | 1,4 (0,7) | 1,6 (0,8) |
